# Radar-Based Monitoring: A Proof of Principle Study in a Piglet Model for a Novel Approach in Non-Contact Vital Sign Monitoring †

**DOI:** 10.3390/s26072139

**Published:** 2026-03-30

**Authors:** Sybelle Goedicke-Fritz, Daniel Schmiech, René Thull, Elisabeth Kaiser, Christina Körbel, Matthias W. Laschke, Aly Marnach, Simon Müller, Erol Tutdibi, Nasenien Nourkami-Tutdibi, Regine Weber, Michael Zemlin, Andreas R. Diewald

**Affiliations:** 1 Department of General Pediatrics and Neonatology, Saarland University, Campus Homburg, 66421 Homburg, Germany; 2 Laboratory of Applied Radar Technology and Optical Systems (LaROS), Trier University of Applied Sciences, Schneidershof, 54293 Trier, Germany; 3 Institute for Clinical and Experimental Surgery, Saarland University, 66421 Homburg, Germany

**Keywords:** non-contact monitoring, neonatal intensive care unit (NICU), preterm infants, breathing rate, radar, piglets

## Abstract

(1) Background: Hospitalized preterm infants often require months of vital signs monitoring in the neonatal intensive care unit. Today, wired sensors are essential for survival, but are associated with numerous disadvantages including sensor dislocations, skin trauma and hygiene risks. Non-contact vital sign monitoring would therefore represent a significant improvement in the care of hospitalized neonates. (2) Objective: This study aims to lay the foundation for non-contact radar-based monitoring of the respiratory rate, which could be used in the neonatal intensive care unit. (3) Methods: We developed a radar-based vital parameter monitoring system for recording the respiratory rate of premature infants in a pediatric incubator. The novel system employs a four-channel I/Q FMCW radar with compact, application-specific antennas optimized to cover the defined area of interest on the infant’s thorax. As a proof-of-principle study, the system was tested in six anesthetized newborn piglets. (4) Results: Using the radar-based system, thorax movements were detected and the respiratory rate was calculated. We observed a high accordance between the signals of respiration detected by the novel radar sensor with the signals of the cable-bound monitor in resting piglets. (5) Conclusions: The novel radar sensor is suited for measuring respiration in the piglet model. In future, the sensor should be optimized in order to improve its robustness against disturbances body movements and in order to allow detection of heartbeat.

## 1. Introduction

Prematurity remains one of the major global health challenges, as approximately 10% of all children are still born prematurely worldwide, with a rising incidence [1,2]. Nowadays, the ability to treat preterm infants at the limit of viability, starting at a gestational age of 22 to 23 weeks, is a challenging task for every neonatal intensive care team [3,4]. These vulnerable patients are exposed to a wide range of short- and long-term morbidities. Many risk factors of preterm infants and their associated short-term morbidities can be reduced by standardization of therapy regimens, regionalization, treatment in level III centers, and close cooperation between affiliated disciplines, resulting in improved pre- and postnatal care [5,6]. The risk factors influencing mortality and morbidity of preterm infants, as well as their long-term morbidities, have been well described and analyzed in various longitudinal cohort studies [7,8,9].

During treatment in the neonatal intensive care unit (NICU), preterm infants are monitored closely and continuously [10,11]. Daily evaluation and changes in the general condition of these vulnerable patients are based on vital signs such as heart rate, breathing frequency and patterns, and temperature, in combination with clinical examination. Additional diagnostics such as laboratory work and sonography are performed if necessary. Slight deviations in breathing pattern and heart rate, such as bradycardia and apnea, can be early prodromal signs of infection. Differentiating these prodromal signs from expected physiological patterns in immature infants, such as bradycardia–apnea syndrome or respiratory deterioration due to lung immaturity, is challenging and requires experience. The interpretation of changes in vital signs remains a cornerstone of neonatal treatment and monitoring. Hence, methods to improve vital sign monitoring are the focus of current research.

### 1.1. Current State of the Art in Vital Sign Monitoring of NICUs

Currently, monitoring of heart rate and rhythm is performed via electrocardiography (ECG) and blood oxygen saturation (SpO_2_) using pulse oximetry based on photoplethysmography (PPG) [12,13,14,15,16]. Both techniques are non-invasive but require contact, as PPG sensors must be placed on the limbs while ECG electrodes need to be attached to the chest wall, regardless of gestational age. Attaching and re-attaching electrodes and sensors can cause irritation and injuries, such as skin ulcers, especially in very low birthweight (VLBW) infants (<1500 g) and extremely low birthweight (ELBW) infants (<1000 g) [1,2,11,12,17,18,19]. Even minor skin injuries can serve as entry points for bacteria and viruses, causing severe infections or sepsis in these vulnerable patients. Moreover, electrodes, plasters, and cables interfere with freedom of movement, particularly during “Kangaroo Care” and therapeutic positioning [14,20,21]. Additionally, certain sensors for premature infants are prone to loss of contact, reducing measurement reliability and triggering false alarms, causing stress and alarm fatigue for staff.

### 1.2. Advantages of Non-Contact Methods

Overall, there are many disadvantages to contact-based vital sign monitoring, making the development of contactless approaches an important step toward improved neonatal care [4,9,11,18,22,23,24]. Non-contact diagnostic methods have been investigated and developed over several years to evaluate their clinical applicability. Some studies focus on detecting biomarkers in metabolic products, e.g., via an electronic nose or ion mobility spectrometry, potentially reducing painful blood sampling [25,26]. Other studies address non-contact vital sign monitoring, which is particularly desirable in neonates to avoid adhesive sensors that can damage fragile skin and even lead to infections or pressure necroses [27,28]. Non-contact monitoring systems based on optical sensors or PPG have been tested for clinical applications [10,29,30,31,32,33]. Optical methods allow contactless monitoring of heart rate, respiration, or oxygen saturation, but require visible skin. Items like clothing, bed covers, or incubator lids hinder their use, and variations in skin color or hairiness complicate data analysis [10,29,34,35,36]. Cable-reducing approaches for monitoring vital signs, including respiration and heart rate, are promising, but adhesive pads remain necessary for fragile neonatal skin [14,15].

### 1.3. Radar-Based Vital Sign Monitoring

Radar-based non-contact monitoring of cardio-respiratory activity has been investigated extensively over recent years in both human subjects and animal models, including prior RaMoSS-related studies and provides advantages over alternative methods. Radar measurements have already been demonstrated in pediatric and adult populations, including neonatal intensive care unit (NICU) environments, with a primary focus on respiratory rate extraction and feasibility under clinical conditions [37,38,39]. Radar-based measurements offer the advantage of not requiring any sensors to be attached to the skin.

Additionally, covering materials such as blankets, incubator lids, or clothing in more mature preterm or term infants do not obstruct signal acquisition [40,41,42,43,44,45,46]. Initial limitations due to bulky and costly waveguide components have been overcome with recent technological advances [47]. These characteristics make radar-based approaches particularly attractive for neonatal monitoring in clinical environments.

### 1.4. Radar Technology for Animal Monitoring

Radar systems have been applied to non-contact vital sign monitoring in animals, primarily as proof-of-concept demonstrations across different species [48,49,50]. These studies confirm technical feasibility, including respiratory and cardiac signal detection. However, most experimental designs were not intended to address neonatal physiology or translational requirements relevant to neonatal intensive care environments. This gap highlights the need for model systems that reflect neonatal-specific monitoring challenges.

### 1.5. Mini-Piglets as an Animal Model

A key challenge in developing a new sensor for neonatal vital sign monitoring is the necessity of validation in vulnerable populations. Adults are easier to study due to their cooperative nature, and prior radar feasibility studies have mostly been conducted on adults in laboratory settings [45,51,52,53]. Independently of radar sensing, minipig models have been widely adopted in neonatology-related research due to their close similarity to human neonates with respect to body size, thoracic mechanics, and cardio-respiratory parameter ranges, making them particularly suitable for translational investigations preceding studies in preterm infants [54,55,56,57,58].

Piglets have been successfully included as animal models in studies for pediatric research in the past [59,60]. The Aachen minipig has a comparable phenotype to the Göttingen minipig, which is often used as a laboratory animal model [61]. Minipigs have lower absolute and relative daily gains than fattening pigs, with an average weight of 0.51 kg at birth and 3.58 kg after 4 weeks [62]. Not only in terms of their size and weight, the piglets examined in this study are very comparable to preterm infants, ranging in stage from 28 weeks gestation to maturity but also regarding the measured average respiratory rate [63,64,65]. The lung-to-body ratio is very similar between human and porcine neonates.

According to Sipos et al. the mean respiratory rate during the first three weeks of life was approximately 80 to 90 breaths per minute, with minor mean fluctuations [66]. Older reference values indicate 34 to 64 breaths per minute as physiological in conscious suckling piglets [67]. It should be taken into account that these values refer to conventionally kept fattening pig breeds. Importantly, neonatal physiology exhibits distinctive characteristics, including higher resting respiratory and heart rates, altered chest wall compliance, and unique patterns of cardio-respiratory coupling compared to older children and adults. These traits impose specific challenges for non-contact sensing modalities and necessitate model systems that replicate these demands. Recent studies using neonatal animal models have underscored the value of minipigs for replicating human preterm physiology, including lung development trajectories and oxygenation responses relevant to NICU monitoring paradigms [68,69,70,71].

### 1.6. Technical Overview

The monitoring system used in this study is based on a four-channel frequency-modulated continuous wave (FMCW) radar operating in the 24 GHz ISM band. A frequency-modulated chirp is transmitted toward the thoracic region, where respiratory motion produces displacement-dependent phase variations in the reflected signal. Multi-channel in-phase and quadrature (I/Q) reception preserves phase information and improves motion sensitivity. Subsequent signal processing extracts low-frequency components associated with respiration while suppressing static clutter.

This architecture enables continuous, contact-free detection of thoracic motion and forms the technical basis for the neonatal monitoring application investigated here.

### 1.7. Aim of This Study

The aim of this study was to investigate whether radar-based, contact-free monitoring can be meaningfully adapted to physiological conditions relevant for neonatal intensive care. A minipig model with sedated animals was employed to create an experimental setting that reflects key characteristics of preterm infants and allows systematic evaluation under clinically oriented conditions. The work focuses on assessing the feasibility, reliability, and clinical relevance of radar-derived respiratory signals in this neonatal-representative model. By combining radar sensing with a physiologically appropriate animal model, the study addresses an unresolved step between technical feasibility studies and future neonatal applications. Thus, the primary objective is to establish a translational basis for subsequent validation in preterm infants.

## 2. Material and Methods

### 2.1. Animals

In preparation for the experiments, the pregnant sow (Aachen Minipig, Heinrichs Tierzucht GmbH, Heinsberg, Germany) was housed in the animal husbandry with a special litter bay three weeks before the expected date of delivery. After spontaneous birth, the weaning piglets had constant access to water and a warming lamp. Nine piglets (Aachen Minipig) from one litter were included in the study, at the age of one to four weeks.

To minimize motion artifacts piglets were sedated during measurements. When anesthetized piglets are used as models for vital sign monitoring of awake or sleeping neonates, anesthesia-related effects on reference values should be considered (e.g., propofol has been associated with a decrease in mean arterial pressure in humans [72]). Although anesthesia may influence physiological parameters, previous studies demonstrate hemodynamic stability during total intravenous anesthesia with propofol and fentanyl in piglet models [73], supporting controlled respiratory monitoring conditions.

Sedation was induced with 4% isoflurane in oxygen via a face mask to place a peripheral venous catheter in one of the ear veins. Blood was drawn from the catheterized vein through a three-way stopcock for blood gas analysis. Anesthetic maintenance was achieved by anesthetic depth-controlled IV administration of propofol (2–3 mg/kg/h). Moderate sedation was necessary to reduce movement artifacts and allow detection solely of breathing and heart rate. The spontaneously breathing piglet was placed in the center of the temperature-regulated incubator.

The study was carried out in compliance with ARRIVE guidelines (Animal Research: Reporting of In Vivo Experiments [74]). All measurements were taken under the constant supervision of physicians and veterinarians.

### 2.2. Experimental Setup

**Figure 1:**
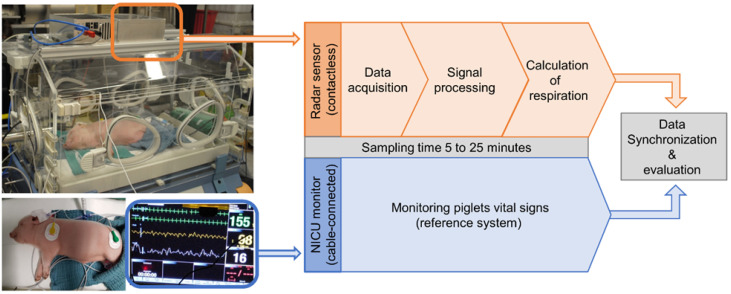
Experimental setup. A piglet with vital parameters similar to a premature infant was placed in an NICU incubator. Pulse, oxygen saturation, and respiration rate were monitored by a camera-based non-contact pulse oximeter (orange) and a conventional cable-connected NICU monitor via pulse oximeter sensor and three-channel ECG (blue).

The experimental setup was designed to allow testing and evaluation of the microwave-based sensor for an innovative NICU application under realistic conditions (Figure 1). A conventional NICU incubator 8000 IC (Drägerwerk AG & Co KGaA, Lübeck, Germany) was used, with the radar system placed on top at approximately 400 mm above the piglet [75,76,77,78].

Vital parameters were simultaneously recorded by a conventional cable-connected monitor (CARESCAPE B650, GE Healthcare, Chicago, IL, USA) and the radar system. The reference monitor measured oxygen saturation (SpO_2_) using red and infrared light and thoracic ECG. Measurements lasted 5 to 25 min per session, with multiple sessions per test day. Respiration data from both systems were synchronized and evaluated.

### 2.3. Principles of Radar Sensor System and Impedance Pneumography

**Figure 2:**
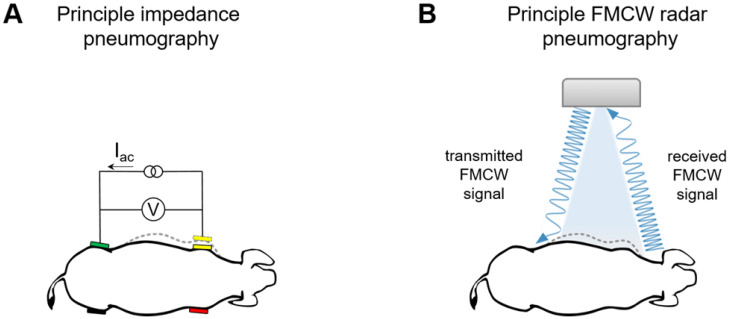
Principle of conventional NICU monitoring system and radar. (**A**) Impedance pneumography; (**B**) 24 GHz FMCW radar.

Impedance pneumography detects breathing by measuring small changes in electrical impedance across the thorax. A high-frequency, low-amplitude sinusoidal current is injected, and the resulting voltage changes correspond to thoracic expansion/contraction (Figure 2A) [79,80].

The radar-based sensor (RaMoSS) is a four-channel frequency-modulated continuous wave (FMCW) system operating at 24 GHz (ISM band). It measures thoracic displacement non-invasively from a fixed position above the incubator. Continuous frequency modulation allows detection of distance to moving or static objects, providing a phase signal proportional to chest motion (Figure 2B).

### 2.4. Radar Monitoring Sensor System (RaMoSS)

**Figure 3:**
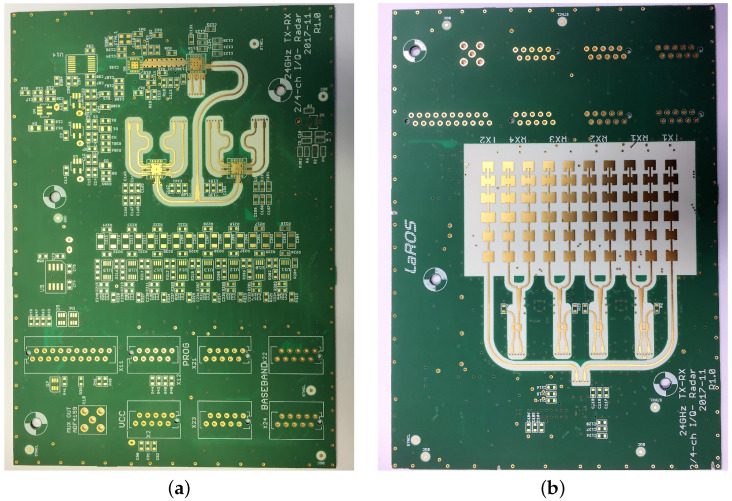
Final PCB design of the radar system: (**a**) top side with RF front end and signal processing components, (**b**) bottom side with integrated antenna arrays.

**Figure 4:**
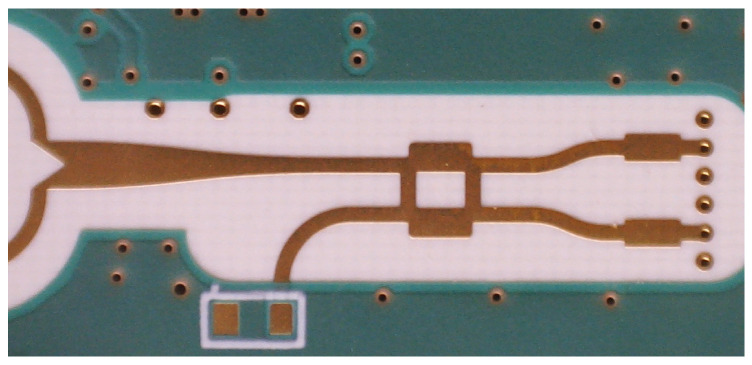
Design of the branch–line coupler.

The RaMoSS radar operates at 24.125 GHz with a 250 MHz bandwidth. The PCB integrates two transmitting and four receiving channels for spatial diversity, connected to series-fed patch antenna arrays optimized for broadside radiation and coverage of the thoracic region. Active RF components are placed on the top layer, antennas on the bottom, with interconnections via impedance-matched microstrip vias. The system uses a passive 3 dB branch-line coupler per receiver to generate I/Q signals, forming the input for displacement and respiratory motion estimation (Figure 3 and Figure 4).

Further details of this printed circuit board (PCB) are described in [75] and summarized in Table 1.

The radar front end was specifically designed for application in neonatal incubators, where limited space, mechanical constraints, and electromagnetic compatibility requirements demand a compact and integrated sensor solution. Therefore, all radio-frequency (RF) components, including transmitting and receiving antennas, mixers, and signal distribution networks, were realized on a single four-layer printed circuit board (PCB). The operating frequency of 24.125 GHz was selected within the industrial, scientific, and medical (ISM) band to allow continuous operation without interference with medical equipment. Due to the wavelength of λ=12.5 cm and the ramp repetition frequency of 32 Hz, the radar system is able to detect a maximum target speed of 1 $\frac{m}{s}$.

The compact design enables mounting of the sensor unit on the top of the incubator, centered above the mattress, while ensuring a defined radiation footprint limited to the region of interest. This footprint was optimized to cover the thoracic area of the piglet while suppressing reflections from surrounding incubator structures, thereby improving the signal-to-noise ratio.

The antenna structures were implemented on the bottom layer of the PCB, while active RF components such as frequency synthesizers, amplifiers, and mixers were placed on the top layer to simplify assembly and thermal management.

The underlying antenna concept is based on a six-patch series-fed traveling-wave microstrip antenna array, which was specifically designed for operation in the 24 GHz ISM band and optimized with respect to impedance matching, bandwidth, and radiation footprint, as described in detail in [81].

Figure 3 illustrates the top and bottom sides of the final PCB. The top side mainly contains the RF front end, including the local oscillator distribution, mixers, and intermediate frequency signal routing. The bottom side hosts the antenna arrays and associated feed networks. Vertical interconnections between the layers were realized using impedance-matched microstrip vias to ensure minimal reflection losses within the 24 GHz bandwidth.

To generate complex baseband signals required for precise displacement detection, each receiving path employs a passive 3 dB branch–line coupler to generate in-phase (I) and quadrature (Q) components. This approach avoids the need for more complex and power-hungry I/Q mixer architectures while maintaining phase stability across all channels. The resulting I/Q signals form the basis for subsequent range and motion estimation.

**Figure 5:**
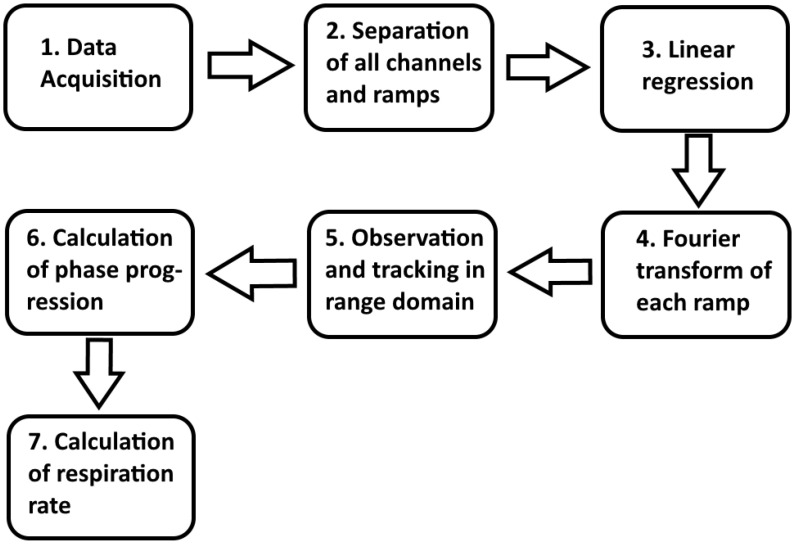
Signal processing structure.

The signal processing structure of the radar monitoring sensor system (RaMoSS) in this pilot study on piglets is shown in Figure 5.

### 2.5. Radar Signal Processing

Although the implemented FMCW radar operates with a bandwidth of 250 MHz, resulting in a range resolution of approximately 0.6 m, the respiration monitoring in this study does not rely on range resolution. Instead, respiratory motion is extracted from phase variations within a predefined area of interest. Due to the high phase sensitivity of FMCW radar, small periodic chest displacements in the sub-millimeter range can be reliably detected, enabling accurate respiration monitoring despite the coarse range resolution. The transmitted FMCW signal reflects off the infant’s chest and is received by four I/Q channels.

The received signals are converted to baseband and processed to obtain range-resolved phase information: The recorded raw data signals are assigned to their corresponding receiving channels and split into the different frequency ramps. Then, a signal correction in the form of a linear regression is performed to extract unintentional signal components in the close range. The corrected residual signal is transformed to the range domain in order to detect the target in the range-of-interest. The phase information at the target position is evaluated separately for each radar channel and is combined afterwards to represent the breathing motion between sensor and patient. Respiratory motion is extracted from low-frequency phase variations, while higher frequency components correspond to heartbeats. This pipeline allows differentiation of vital sign signals from static clutter and provides a real-time assessment of respiratory rate.

## 3. Results

This section presents the measurement sequences from six piglets used to evaluate the novel non-contact radar-based monitoring system (RaMoSS). RaMoSS represents an innovative approach as it simultaneously combines four-channel FMCW radar, integrated series-fed patch antenna arrays, and I/Q-based signal processing to detect respiration without any skin contact. These results illustrate both the system’s feasibility and its potential advantages in comparison to conventional NICU monitoring systems.

### 3.1. Piglets as Suitable Animal Models for Human Preterm Neonates

Piglet respiration was monitored using the non-contact RaMoSS system and simultaneously by impedance pneumography via a conventional monitor system commonly used in NICUs. The animals were examined at regular postpartum time intervals. The respiratory data presented here are from piglets between their 5th and 19th day of life, which corresponds to the human neonatal period and covers the piglets’ suckling phase. In terms of size and weight at the time of measurement, the piglets in this study are very comparable to a wide range of (premature) patients treated in a neonatal intensive care unit (Table 2).

Piglets 2 and 6, with weights of 1250 g and 1050 g, respectively, correspond to preterm infants of the very low birth weight (VLBW) group. Piglets 4 and 5, with 1900 g and 2210 g, respectively, correspond to the low birth weight (LBW) preterm infant group. Movement patterns and the occurrence of transients in the sedated piglets were also very similar to those of preterm infants. The measured average respiratory rate of the piglets was comparable to that of preterm infants, showing a dependence on age and weight: the mean respiratory frequency decreased with increasing age. For these reasons, Aachen Minipigs are highly suitable as animal models for preterm infants [66].

### 3.2. Radar-Based Monitoring Capable of Detecting Motion States and Apnea Episodes

**Figure 6:**
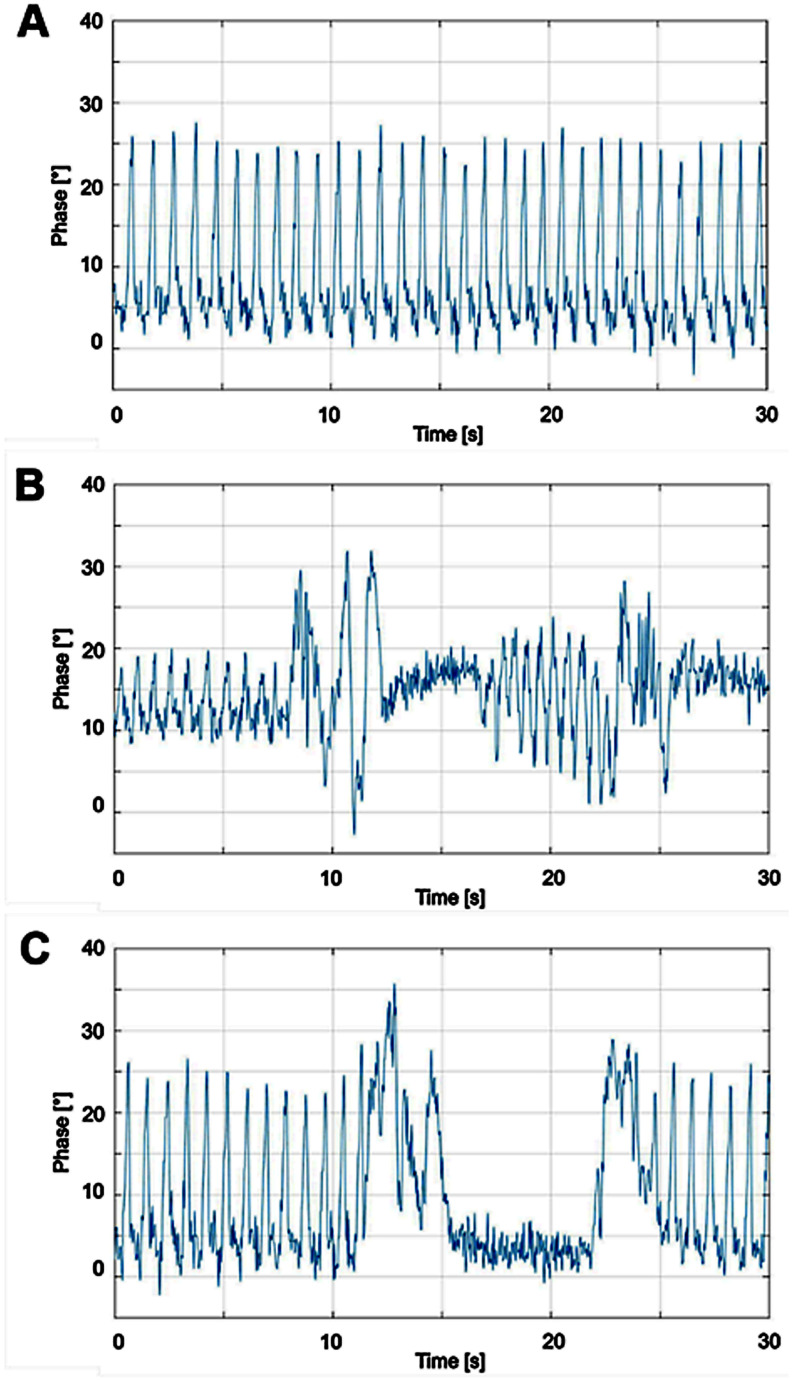
Radar-based sensor system: phase progression of piglet breathing data. Exemplary sections for phase progression of three piglet breathing measurements: (**A**) Piglet 2, completely calm, no strong movements. (**B**) Piglet 6, calm behavior in the beginning (0–8 s) with strong movement episodes (8–30 s). (**C**) Piglet 2, calm behavior with apnea episode (11–25 s). See Table 2 for more information on individual measurements and piglets. The corresponding breathing cycles, compared to the reference monitor in a 1000 s window, are shown in Figure 7.

Phase progression under different piglet conditions is presented in Figure 6, showing a 30 s window for each measurement. The phase diagrams form the basis for further radar data processing to calculate the respiratory rate and assess measurement quality. The periodic waveform in Figure 6A originates from a sleeping, low-motion piglet, representing respiration without disturbances. Irregular waveforms, shown in Figure 6B, correspond to movement-intensive states, such as wake-up phases. Figure 6C shows a transition from periodic to irregular patterns, followed by a temporary decrease in breathing over 14 s, consistent with a real observed respiratory interruption. The RaMoSS system, with appropriate data processing, reliably detects apnea episodes.

The phase progression diagrams in Figure 6 illustrate how RaMoSS converts I/Q signals from the four receiving channels into range-resolved phase information. Low-frequency variations correspond to respiratory motion, while higher-frequency components indicate cardiac activity. This approach allows the system to separate vital signs from static clutter and transient movements, highlighting its innovative ability to detect subtle respiration patterns even in small preterm-like subjects. It should be noted that the focus of this study is on respiration monitoring and respiration-related artifacts under sedation, rather than heartbeat detection. Apnea episodes are identified by the absence of periodic respiratory phase modulation rather than by irregular cardiac activity, while body motion can temporarily obscure fine physiological signals, the differentiation between apnea and gross movement is based on the loss of regular respiratory patterns. A detailed analysis of heartbeat extraction during apnea episodes is beyond the scope of this work and will be addressed in future studies.

### 3.3. Radar System as an Alternative to Cable-Based Reference Monitoring

**Figure 7:**
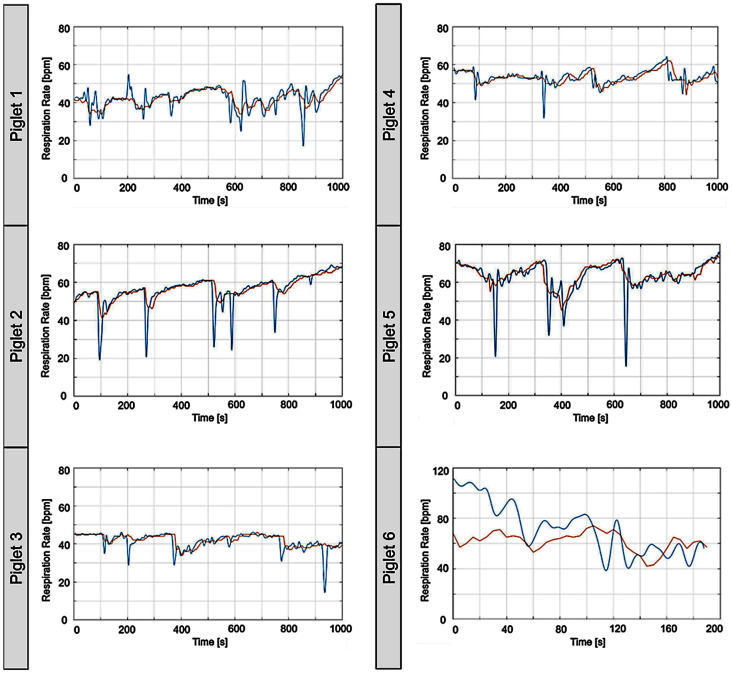
Breathing rate comparison over time recorded by cable-free radar-based sensor and cable-based reference monitor. As an example, calculated breathing cycles of six different piglets (Table 2) are shown over a period of 1000 s each. Respiratory data were simultaneously recorded using the cable-connected reference monitor commonly used in the NICU (red) and the radar-based non-contact monitoring system (blue). Time synchronization and plotting were performed using MATLAB version 9.5. Piglets 1–5 were in a physical motionless state during the measurement period. Measurement of piglet 6 was stopped after 5 min due to high-intensity movement.

The simple signal processing structure in Figure 5 provides breathing signals from the radar system. Time-synchronized representation in Figure 7 allows direct comparison between the respiration curves from the conventional cable-connected reference monitor and the cable-free radar system. Raw data from both platforms were extracted, synchronized using MATLAB (MathWorks, Inc., Natick, MA, USA), and further analyzed.

**Figure 8:**
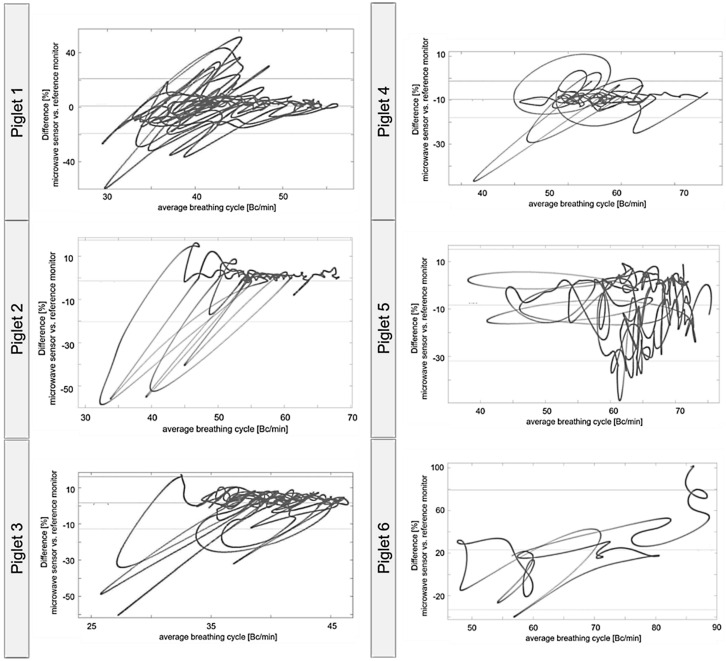
Comparison of relative differences in respiration rate measured with the cable-free radar-based sensor and the cable-based reference monitor. Measurements of respiration of six piglets are presented (Table 2). Shown are the relative differences in simultaneously recorded data as a function of the mean breathing rate per minute. Data correspond to those shown in Figure 7. Piglets 1–5 were motionless during a 25 min measurement period. Measurement of piglet 6 was stopped after 5 min due to high-intensity movement.

Piglets 1–5 were mostly in a low-motion state, and respiration data from both systems showed high comparability, as confirmed by correlation coefficients (Table 2). Respiratory interruptions observed by RaMoSS were not, or only partially, captured by the reference monitor, which explains strong deviations at very low respiratory frequencies in the Bland–Altman diagrams (Figure 8). These interruptions were real, observed events, not artifacts of the radar system. When piglets moved extensively, the simple signal processing was insufficient, as seen with piglet 6. Here, RaMoSS measurements deviated more strongly, while the reference monitor was more robust to movement, resulting in higher standard deviations and relative differences.

Table 2 and Figure 7 and Figure 8 not only compare measured respiration rates but also reflect the robustness of the simple signal processing pipeline described in Section 2.4. Nevertheless, for low-motion conditions, the radar system demonstrates high dynamic responsiveness and effectively captures vital respiratory patterns. Compared to conventional impedance pneumography and cable-based monitors, RaMoSS provides continuous, non-contact respiratory monitoring and is sensitive enough to detect brief apnea episodes that are often missed by standard devices. This demonstrates the added value of the radar-based approach in preterm neonatal care simulations.

## 4. Discussion

The aim of this project was to establish a microwave-based system in preparation for its implementation in NICUs. Premature neonates often require prolonged stays in intensive care units after birth, during which their vital parameters must be continuously monitored. Conventional methods achieve this using multiple cable-based sensors [18,23,82]. In contrast to previous feasibility studies, the present work focuses on a fully integrated FMCW radar system specifically designed for neonatal incubator environments and evaluates its performance under clinically realistic conditions using a preterm-relevant animal model.

Radar technology offers the possibility of detecting respiration through abdominal displacement relative to a permanently installed sensor, e.g., above the bed. Even subtle positional changes can be detected and processed by the system. The RaMoSS system presented here enables non-contact monitoring of respiratory rate, reducing stress and skin irritation. In NICUs, a non-contact system could decrease staff–patient interactions and address the limitations of current cable-based systems.

In this study, the principle functionality of the 24 GHz FMCW radar sensor, RaMoSS, was demonstrated on a conventional NICU incubator. The respiratory rate of a piglet, whose vital parameters resemble those of neonates, was monitored using RaMoSS and a conventional cable-connected reference monitor.

Phase progression assessment, as a primary data set of radar-based vital sign monitoring, is a very useful feature, for example, in long-term respiratory monitoring. Using these data, the data quality of the recorded signals can be easily checked, e.g., the presence of irregular respiratory patterns or measurement artifacts. This option is not available to users of currently available reference monitors.

In this study, the RaMoSS showed very good results for piglets in low-motion conditions. In this first proof-of-principle test, it was desirable that the physical behavior of the piglet did not change during a measurement. Movements of the animal influence the radar signal, which is primarily intended to detect vital signs in a stable manner. During the study, some measurements were conducted under ideal conditions, with the piglet sleeping calmly, allowing stable respiration monitoring by RaMoSS. More frequently, the piglet moved its feet or head, sometimes multiple independent movements occurring simultaneously. In the sleeping state, the sensor displayed a moderate to high correlation with values measured by the reference monitor. However, this result must be interpreted considering several aspects:

First, since the system acquires signals based on piglet motion, movement proved to be an interfering variable for the correct detection of respiratory rate. This limitation must be addressed in the next step, even though RaMoSS will ultimately be applied primarily for monitoring sleeping neonates. The signal processing performed here enables respiration detection in a healthy, sleeping piglet.

Second, when the piglet moved, the signal processing was insufficient. Changes in peak detection parameters (e.g., peak prominence) may improve respiratory rate calculation. This could be part of methodological optimization and further application studies. Since only the in-phase (I) signals of the radar were used in this project, the next step will be the complete use of the quadrature signals (I+jQ), which is expected to improve results, especially in low-frequency ranges. Random body movement remains a major challenge in non-contact vital signs monitoring systems. To obtain better results, the exact position of the target object, primarily the thoracic motion in this study due to breathing, must be extracted.

From a hardware perspective, future iterations of the RaMoSS will benefit from more focused spatial selectivity, for example by exploiting the multi-channel antenna configuration for digital beamforming or range–angle discrimination to suppress motion originating from extremities. On the signal processing level, combining range-gated phase analysis with full complex I/Q evaluation and adaptive motion artifact detection is expected to improve the reliable separation of respiratory motion from gross body movements. Additionally, data-driven classification approaches could be employed to distinguish genuine vital sign patterns from non-respiratory motion artifacts in real time.

Third, apnea episodes of the piglets were observed during the measurements. Such respiratory interruptions are also very common and physiological in neonates, known as apnea of prematurity [83,84]. In the measurement series, these episodes were detected very sensitively by the radar sensor system in almost all piglets (see Figure 6C as example) and were verified as true apnea by personal observation. In contrast, these apnea episodes were not or only barely detected by the conventional NICU monitor, which monitors vital signs via a cable connection to the patient. Since the radar detects the apnea episodes while the reference monitor does not, there are direct deviations in the correlation analysis.

In the Bland–Altman diagrams, these deviations are exactly in the areas of low respiratory frequency. After a delay of a few seconds, the reference monitor shows a slight decrease in the detected respiratory rate, likely due to the lower sampling rate and wider time window for measurement, calculation, and filtering. Consequently, short apneas cannot be detected to the same extent as with the radar system. Phase progression provides additional information that can be easily obtained and processed for reliable interpretation.

Fourth, it should be noted that the sensitivity of the radar-based monitoring system is both a strength and a weakness. During episodes of piglet movement, respiration data differ between the two measurement methods. In these cases, the movement of the whole body or extremities should be considered as an artifact of the respiration measured secondarily as abdominal motion. To assess the results of the study, it is important to note that the piglets were primarily in the lateral position during the measurements. During their sleep phases, newborns sometimes adopt the lateral position, but mostly the supine or prone position. Especially in the prone position, movement of the extremities is likely less pronounced, causing minimal disturbance to RaMoSS. This circumstance could be considerably less pronounced in adult applications. A young child has a much smaller body surface area compared to an adult. In the signal field of the sensor monitoring an adult thorax, the same setup monitors the entire child, including the most likely moving extremities. In this case, extremity movements directly result in artifacts in the respiratory rate curves. Therefore, the RaMoSS would be markedly less critical to use on an adult.

In general, even the use of adhesive pads on the skin, which is critical in children, has less impact in adults. Here, the major advantage of RaMoSS is that hospitalized patients often have very high cable density with continuous monitoring of vital signs, which it is desirable to reduce. In addition, the skin of very old patients is often fragile, leading to similar problems as in neonates regarding the use of adhesive connections in intensive care. For these reasons, it is highly worthwhile to develop continuous vital signs monitoring in a way that does not require a direct connection to the patient. Reducing the cable load could lead to stress reduction, especially for premature and newborn infants in pediatric intensive care units, which would positively impact their recovery.

Nevertheless, there are numerous other applications of an appropriate non-contact sensor system for monitoring vital signs, particularly respiration, beyond its use in pediatrics. Thus, it can be demonstrated that the radar-based sensor system is even superior to the reference system in the case of a sleeping piglet. The study presented here lays a solid foundation for the future application of this technology.

## 5. Conclusions

The present work builds explicitly on prior developments but addresses a distinct and previously insufficiently explored research question. Importantly, this manuscript does not claim novelty in the radar sensing principle itself, nor does it claim the first application of radar measurements in animals or neonate-related contexts. Instead, the innovation lies in the deliberate translational integration of radar-based non-contact vital sign monitoring with a neonatal-representative minipig model, explicitly selected to serve as a preparatory bridge toward future NICU deployment in preterm infants.

While radar measurements have been conducted in children, in animals, and in neonatal clinical settings separately, their targeted combination for clinically motivated validation has not been systematically addressed in previous radar-based studies or in the broader radar vital sign literature.

Furthermore, the present study aligns with emerging translational frameworks in biomedical sensing, where rigorously characterized animal models are positioned as essential intermediates before first-in-human neonatal trials, particularly for technologies that must deliver continuous, contact-free monitoring without interference from movement, humidity, or caregiving activities typical of NICU environments [85,86].

This translational emphasis is critical for establishing physiological validity and deployment readiness ahead of studies in human neonates. By employing minipigs as a surrogate model for preterm infants, based on comparable body dimensions and physiologically relevant ranges of respiratory and cardiac activity, this study establishes a validation framework that is explicitly aligned with known NICU requirements. The experimental protocol, sensor placement, and analysis strategy are designed to evaluate accuracy, robustness, and physiological consistency of radar-derived vital parameters under conditions representative of future neonatal monitoring scenarios.

In this sense, the present work closes a critical translational gap between prior feasibility-focused radar investigations and the stringent functional demands of continuous, non-contact monitoring in neonatal intensive care. This clinically oriented positioning, rather than the introduction of a new sensing modality, constitutes the primary contribution and novelty of the manuscript.

## 6. Outlook

The study demonstrates the general feasibility of non-contact vital sign monitoring in a clinical pediatric environment. However, the limitations in the applicability of the RaMoSS system, as highlighted in this study, indicate a clear need for methodological optimization. Any non-contact system must reliably differentiate between genuine vital signs and artifacts caused by movements, such as limb motion.

Future developments of the sensor should aim to improve robustness against such artifacts. A follow-up study is warranted to optimize the data collected in this proof-of-principle study. This should include enlarging the dataset and extending measurements from piglets to premature and newborn infants. These very young pediatric patients constitute a significant target group for non-contact, non-invasive diagnostic methods. Increasing the sample size should be accompanied by technical improvements to the sensor system to maintain high-quality monitoring under suboptimal measurement conditions.

For clinical application, it will also be important to determine thresholds for respiratory pauses that warrant alarm activation. Excessively sensitive detection of short, physiological apneas could result in unnecessary alarms. In prospective implementations of radar-mediated monitoring systems with alarm coupling, such parameters would need careful programming and validation. Another future goal is to expand non-contact monitoring to additional vital parameters, such as heart rate and oxygen saturation, especially in premature infants, since apnea events are often accompanied by bradycardia [28]. Beyond neonates, follow-up studies could also target larger children and adult populations.

Despite current methodological limitations, this study provides a strong foundation for further improvements in signal processing for non-contact vital sign monitoring.

## Figures and Tables

**Table 1:** Parameters of the radar system.

Waveform	FMCW
Sample points per ramp	128
Sampling frequency	8192 Hz
Ramp repetition rate	32 Hz

**Table 2:** Piglet data.

Piglet	1	2	3	4	5	6
Sex	female	female	female	male	female	female
Weight [g]	2520	1250	2710	1900	2210	1050
Age [days]	19	6	19	11	19	5
Radar vs. reference (respiration rate)						
$R^{2}$	0.7948	0.7788	0.6807	0.6607	0.5809	0.3688
Mean	0.88	−1.36	−0.84	0.42	−8.35	23.00
CI 95%	−19.25	−20.23	−15.28	–	−31.93	−33.16
	21.00	17.51	13.61	–	15.24	79.16
Intern	513	711	713	812	413	441

## Data Availability

The program code used in this study cannot be disclosed as it is proprietary to ELABS AG, Germany and is subject to confidentiality agreements. The data utilized is owned by CUBICAL GmbH, Germany; it is not available for public disclosure. Both ELABS AG and CUBICAL GmbH were participants in the BMBF-funded project.

## Abbreviations

The following abbreviations are used in this manuscript:

ARRIVE	Animal Research: Reporting of In Vivo Experiments
CI	Confidence interval
ECG	Electrocardiography
ELBW	Extremely low birth weight
FMCW	Frequency-modulated continuous wave
GHz	Gigahertz
I/Q	In-Phase and quadrature
ISM	Industrial, Scientific and Medical (band)
LBW	Low birth weight
MATLAB	Matrix Laboratory (MathWorks, Inc.)
NICU	Neonatal intensive care unit
PCB	Printed circuit board
PPG	Photoplethysmography
RaMoSS	Radar monitoring sensor system
SpO_2_	Peripheral oxygen saturation
VLBW	Very low birth weight
